# Characterization of Early Stage Parkinson's Disease From Resting-State fMRI Data Using a Long Short-Term Memory Network

**DOI:** 10.3389/fnimg.2022.952084

**Published:** 2022-07-13

**Authors:** Xueqi Guo, Sule Tinaz, Nicha C. Dvornek

**Affiliations:** ^1^Department of Biomedical Engineering, Yale University, New Haven, CT, United States; ^2^Department of Neurology, Yale School of Medicine, New Haven, CT, United States; ^3^Department of Radiology and Biomedical Imaging, Yale University, New Haven, CT, United States

**Keywords:** Parkinson's disease, early-stage characterization, fMRI, long short-term memory, deep learning, functional connectivity

## Abstract

Parkinson's disease (PD) is a common and complex neurodegenerative disorder with five stages on the Hoehn and Yahr scaling. Characterizing brain function alterations with progression of early stage disease would support accurate disease staging, development of new therapies, and objective monitoring of disease progression or treatment response. Functional magnetic resonance imaging (fMRI) is a promising tool in revealing functional connectivity (FC) differences and developing biomarkers in PD. While fMRI and FC data have been utilized for diagnosis of PD through application of machine learning approaches such as support vector machine and logistic regression, the characterization of FC changes in early-stage PD has not been investigated. Given the complexity and non-linearity of fMRI data, we propose the use of a long short-term memory (LSTM) network to distinguish the early stages of PD and understand related functional brain changes. The study included 84 subjects (56 in stage 2 and 28 in stage 1) from the Parkinson's Progression Markers Initiative (PPMI), the largest-available public PD dataset. Under a repeated 10-fold stratified cross-validation, the LSTM model reached an accuracy of 71.63%, 13.52% higher than the best traditional machine learning method and 11.56% higher than a CNN model, indicating significantly better robustness and accuracy compared with other machine learning classifiers. Finally, we used the learned LSTM model weights to select the top brain regions that contributed to model prediction and performed FC analyses to characterize functional changes with disease stage and motor impairment to gain better insight into the brain mechanisms of PD.

## 1. Introduction

Parkinson's disease (PD) is a common and complex neurodegenerative disorder (Bloem et al., 2021), affecting around 9.4 million people around the world in 2020 (Maserejian et al., 2020). According to the Hoehn and Yahr scaling, five stages of disease severity have been proposed for PD (Hoehn and Yahr, 1998). In the early stages, patients only have mild signs affecting one side (stage 1) or both sides (stage 2) of the body. Accurate disease staging is crucial for treatment planning, enrollment in clinical trials, and following disease progression.

In recent years, resting-state functional magnetic resonance imaging (rs-fMRI) has been increasingly used to investigate the brain basis of motor and non-motor symptoms, disease severity, and disease progression in PD (Tinaz, 2021). Many of these rs-fMRI studies used the functional connectivity (FC) within and between neural networks as a potential biomarker of PD pathophysiology (Prodoehl et al., 2014; Engels et al., 2018; Wang et al., 2018), but the results have been heterogeneous.

Some studies have investigated machine learning (ML) approaches in early diagnosis of PD using rs-fMRI data (Zhang, 2022). Model-based techniques such as logistic regression are strongly based on prior statistical assumptions, which may not be applicable to real data with variable dependencies (Gao et al., 2018). Model-free algorithms including support vector machine and random forest are able to adapt to inherent characteristics of the dataset with fewer assumptions (Gao et al., 2018) and outperform traditional model-based classifiers in real-world clinical applications. A support vector machine model trained on randomized logistic regression feature selection was implemented to discriminate cognitive status in PD from connection-wise FC patterns and reached an accuracy of 80.0% (Abós et al., 2017). The support vector machine analysis based on inter-group dynamic amplitude of low-frequency fluctuations in PD was found to have significantly higher classification accuracy in reference to controls (Zhang et al., 2019). The random forest algorithm has been successfully implemented for brain connectivity markers and depression and cognitive impairment in PD (Lin et al., 2020, 2021). A brain network graph analysis using rs-fMRI, which identified PD-associated brain network alterations and achieved an average accuracy of 95%, has also been proposed for diagnostic purposes in PD (Kazeminejad et al., 2017). To date, most current classification work using rs-fMRI data has mainly focused on distinguishing between PD patients and healthy control subjects (Dehsarvi and Smith, 2019; Haq et al., 2020; Vivar-Estudillo et al., 2021), but not on disease progression within early PD cohorts. We think that further characterization of the early stages of PD, e.g., understanding brain differences in stage 1 and 2, is necessary to better understand the mechanism and progression of PD.

Recently, deep learning has been successfully implemented in patient representation learning. Convolutional neural networks (CNNs; Kim, 2017) have been widely applied in medical image analysis cases (Anwar et al., 2018). A CNN analysis based on electroencephalogram (EEG) data showed high accuracy (88.25%) for detection of early PD (Oh et al., 2020). Similarly, a CNN model trained on time-frequency representation of EEG was proposed for detection of PD (Khare et al., 2021). A CNN model was trained on structural MRI data to classify PD and healthy controls by transfer learning, and achieved an accuracy of 88.9% (Sivaranjini and Sujatha, 2020). However, the huge time and computational consumption required for CNN training on 4D fMRI data presents an obstacle for its maturation in clinical practice. Furthermore, the black-box nature of CNN methods is a challenge for model interpretability, which is crucial for model utility beyond classification success. Finally, CNNs are well-suited for processing spatial information, but do not take advantage of the temporal sequence of fMRI volumes. On the other hand, the recurrent neural networks (RNN; Medsker and Jain, 2001) have the capacity to capture the temporal dynamics and execute sequential prediction efficiently. The long short-term memory (LSTM; Hochreiter and Schmidhuber, 1997) unit, a prominent variant of RNN with sophisticated gating mechanisms, is designed to overcome the vanishing gradient problem in long sequences. RNNs and LSTMs have first achieved great success in natural language processing (NLP) tasks (Young et al., 2018), and the medical application is also emerging rapidly. Using rs-fMRI datasets, the LSTM model has been investigated on autism identification (Dvornek et al., 2017) and Alzheimer's disease prediction (Hong et al., 2019). The LSTM network has been successfully implemented on voice samples (Rizvi et al., 2020) and walking patterns (Balaji et al., 2021) in PD, but these studies did not investigate the abnormalities in brain functional connectivity. The combination of CNN and RNN has also been investigated in rs-fMRI analysis for neurological disorders. For schizophrenia discrimination, a multi-scale RNN model combining CNN and RNN was proposed, reaching an accuracy of 83.2% (Yan et al., 2019). In Alzheimer's disease (AD) diagnosis, a spatiotemporal model that combines both convolutional and recurrent components was reported to improve AD classification compared with other state-of-the-art approaches (Wang et al., 2019). In major depressive disorder classification, a temporal adaptive graph convolutional network was proposed on rs-fMRI data and FC mapping, achieving a higher accuracy than other state-of-the-art methods of 73.5% (Yao et al., 2020). Nevertheless, in the field of deep learning, PD early-stage characterization has not been investigated with rs-fMRI datasets.

Currently, most rs-fMRI studies in PD enroll a relatively small number (<50) of patients. Due to the variations in data acquisition and pre-processing pipelines among different institutions, as well as the heterogeneity of PD, current research still lacks reproducibility and accuracy across independent datasets. This is certainly a concern for the data-driven approaches. However, some of these concerns can be alleviated with the landmark open data project in PD called the Parkinson's Progression Markers Initiative (PPMI; Marek et al., 2018). The PPMI has organized the first large-scale and the largest-size public multicenter clinical study to study PD progression, including advanced imaging, biologic sampling, and clinical and behavioral assessments.

In this work, we propose to characterize the early stages of PD using the rs-fMRI data obtained from the PPMI database by applying an LSTM-based model. To the best of our knowledge, this project would be the first use of LSTMs to distinguish the early stages of PD (stage 1 vs. stage 2) using rs-fMRI data. The LSTM model allows for the analysis of the raw time-series data from the brain regions of interest (ROIs), retaining more original imaging information compared to models that predict from the pre-processed FC data. We trained and validated the LSTM model under a 5-times repeated 10-fold stratified cross validation and compared the results with a CNN model and traditional machine learning classifiers that predict from FC measures. We also assessed model validity by measuring the association between model output scores and a continuous measure of motor severity in PD. Finally, we interpreted the LSTM model and highlighted the top brain regions and FC measures that contributed most to the classification of the early stages of PD.

## 2. Materials and Methods

### 2.1. Dataset and Pre-processing

All subject data were carefully selected and extracted from the public PPMI database, noting each subject's sex, age, disease onset side, and disease stage. The original PPMI study was conducted following the Declaration of Helsinki and the Good Clinical Practice (GCP) guidelines approved by the local ethics committees of the 24 participating sites in the US (18), Europe (5), and Australia (1) with informed consent obtained from all the enrolled subjects (Marek et al., 2018). Data from 90 subjects were originally curated, but six subjects were excluded due to poor image normalization or excessive head motion. Thus, a total of 84 age-matched (*p*>0.05, unpaired two-sample *t*-test) subjects who were also matched for sex and disease onset side (*p*>0.05, Pearson's chi-squared test) were selected, including 56 subjects at stage 2 and 28 subjects at stage 1. The demographic information of the cohort is summarized in Table 1. All rs-fMRI images were acquired for 8.5 min with TR = 2,400 ms, TE = 25 ms, flip angle = 80°, matrix = 68 ×66, and FOV = 222 mm. After the first four frames were discarded, each patient's raw rs-fMRI scan contained 206 frames in the 4D sequence (8.24 min), yielding a total of 17,304 frames in the dataset. Each single frame was saved in nifti format.

**Table 1:** The demographic information of the cohort.

**Stage**	**Male**	**Female**	**Age (year; mean ±standard deviation)**
HY1	16	12	58.2 ± 9.5
HY2	41	15	62.5 ± 9.6

Pre-processing was performed using the CONN functional connectivity toolbox v17 (Whitfield-Gabrieli and Nieto-Castanon, 2012). The pre-processing steps included motion correction, outlier detection, normalization to the MNI template, smoothing, ROI extraction, and sequence cropping. Outliers were defined as frame-wise displacement above 0.9 mm or global signal changes above five standard deviations. The head motion data of all the subjects are displayed in Table 2, and no significant differences in head motion measures between stages were found. Each frame was aligned to the AAL-116 atlas (Tzourio-Mazoyer et al., 2002) with functional and structural simultaneous gray matter (GM)/white matter (WM)/cerebrospinal fluid (CSF) segmentation and MNI normalization. A Gaussian smoothing at 8 mm full-width at half-maximum (FWHM) was implemented. De-noising steps included correction for physiological and other sources of noise by regressing out the principal components of the white matter and cerebrospinal fluid signal using the CompCor method (Chai et al., 2012), regression of motion artifacts and outliers, and linear detrending. Global signal was not removed. Finally, data were bandpass-filtered (0.008 Hz < *f* <0.09 Hz) to capture the fluctuations of the blood oxygenation level-dependent signal that typically occur within this frequency range at rest. The mean of the voxel values in each ROI was used as that region's signal. Along the time dimension, each mean ROI time-series was extracted and standardized by dividing by the standard deviation among time frames to represent the relative change.

**Table 2:** Head motion characteristics of the rs-fMRI data (mean ± standard deviation).

**Stage**	**Maximum motion (mm)**	**Mean motion (mm)**	**Outlier scans**
HY1	1.2261 ± 0.8207	0.2481 ± 0.1004	5.3928 ± 5.9587
HY2	1.0343 ± 0.8539	0.2342 ± 0.1039	3.8392 ± 9.0549

Considering the relatively small number of early stage subjects for deep learning training, data augmentation was introduced to help the model generalize better and prevent overfitting. The input time sequences were cropped with a fixed sequence length w = 50 (representing 2 min of imaging) and stride length s = 1 to move along the time dimension of the rs-fMRI series. Thus, for each subject, 156 cropped sequences were acquired, which boosted the sample size to 13,104 in total. This augmentation was applied to all the subjects in both training and testing.

### 2.2. LSTM Model

**Figure 1:**
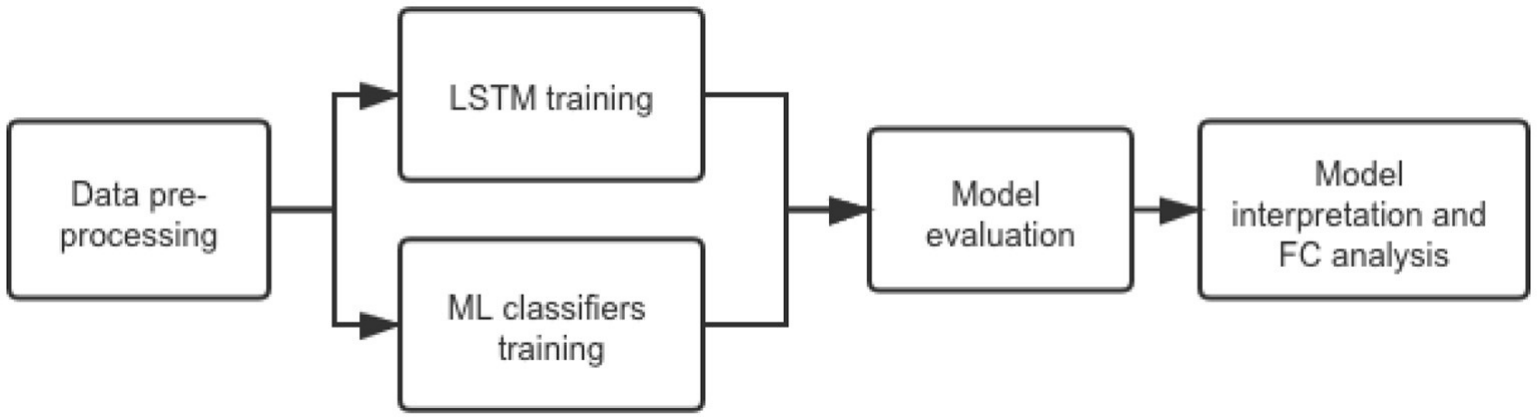
The overall workflow of our project.

We aimed to investigate an LSTM model for PD early stage characterization from rs-fMRI data. We hypothesized that the early stage classification performance would improve compared with traditional ML classifiers that predict from FC measures and CNN-based models that analyze local information. The overall workflow of our project is shown in Figure 1.

LSTMs are a special type of RNN that can address the vanishing gradient and limited long-term memory problems in a vanilla RNN model, taking the previous information and the current data input to update the cell state and hidden state. The key equations in an LSTM cell are:


(1) $$\begin{array}{l} \begin{array}{cc} i_{t}= & \sigma \left(W_{i}x_{t}+U_{i}h_{t-1}+b_{i}\right) \end{array} \end{array}$$

(2) $$\begin{array}{l} \begin{array}{cc} f_{t}= & \sigma \left(W_{f}x_{t}+U_{f}h_{t-1}+b_{f}\right) \end{array} \end{array}$$

(3) $$\begin{array}{l} \begin{array}{cc} \tilde{c_{t}}= & \tanh\left(W_{c}x_{t}+U_{c}h_{t-1}+b_{c}\right) \end{array} \end{array}$$

(4) $$\begin{array}{l} \begin{array}{cc} c_{t}= & i_{t}*\tilde{c_{t}}+f_{t}*c_{t-1} \end{array} \end{array}$$

(5) $$\begin{array}{l} \begin{array}{cc} o_{t}= & \sigma \left(W_{o}x_{t}+U_{o}h_{t-1}+b_{o}\right) \end{array} \end{array}$$

(6) $$\begin{array}{l} \begin{array}{cc} h_{t}= & o_{t}*\tanh\left(c_{t}\right) \end{array} \end{array}$$

where at time step *t*, $x_{t}\in {\mathbb{R}}^{N}$ is the vector of *N* ROI values, $h_{t}\in {\mathbb{R}}^{M}$ is the hidden state, $c_{t}\in {\mathbb{R}}^{M}$ is the cell state, with an input gate $i_{t}\in {\mathbb{R}}^{M}$ deciding what information from the current estimated cell state is updated, a forget gate $f_{t}\in {\mathbb{R}}^{M}$ deciding how much of the previous hidden state should be discarded, and an output gate $o_{t}\in {\mathbb{R}}^{M}$ filtering the cell state to update the hidden state. *W*∈ℝ^*M*×*N*^ is the matrix of weights applied to the input, *U*∈ℝ^*M*×*M*^ is the matrix of weights applied to the previous hidden state, *b*∈ℝ^*M*^ is the bias, and σ is the sigmoid activation function.

**Figure 2:**
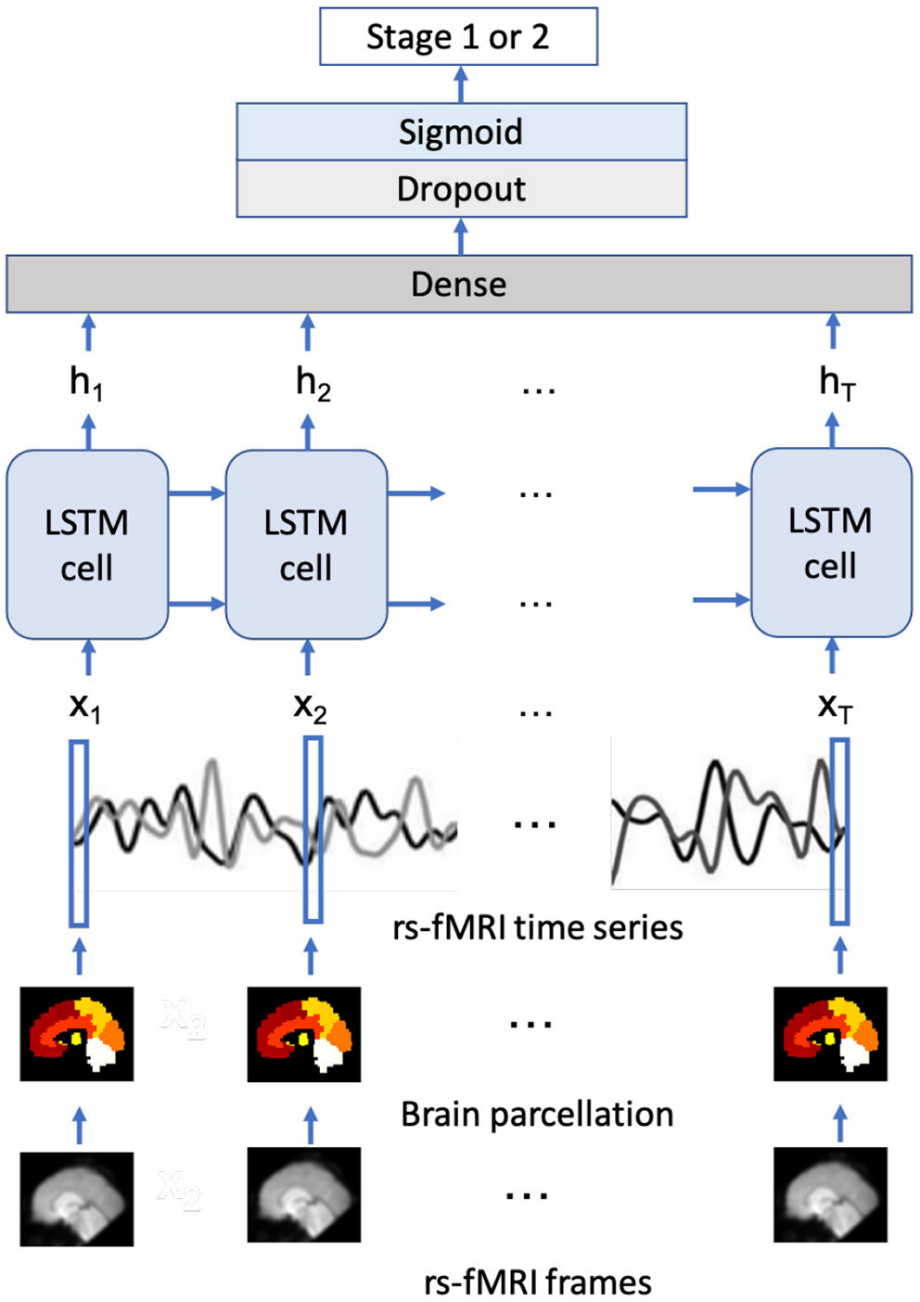
The proposed LSTM model structure.

The proposed LSTM model structure is similar to the model in Dvornek et al. (2017) and shown in Figure 2. The model takes the average time-series of ROIs as the input and then utilizes the output of each time step, which aggregates the decoded hidden state of each cell as the input of the fully connected (dense) layer with 1 node. A dropout layer with dropout rate = 0.5 was integrated between the dense layer and the sigmoid activation layer to prevent overfitting, and the final output of the sigmoid layer would be interpreted as the probability of being assigned as each stage, where higher probabilities correspond to higher likelihood of stage 2. This architecture would directly take the signal at every time point into consideration, which would improve the network's robustness handling noisy rs-fMRI data.

To evaluate the effect of a temporal analysis model, we implemented a CNN model with similar structure as in Oh et al. (2020) on the average time-series ROIs. The CNN model contains three 1-D convolutional layers with stride equal to 1 and kernel size as 7, 5, and 3, respectively. The numbers of the output channels in each layer are 16, 32, and 32, respectively. Following each convolutional layer, a max pooling layer was applied with sliding window size as 2 and stride as 2. The final output was then given by three consecutive dense layers with hidden size as 64, 32, and 1. Similarly, a dropout layer with dropout rate = 0.5 was incorporated after each dense layer to prevent overfitting. The Rectified Linear Unit (ReLU) was applied as the activation function for each layer except the sigmoid activation for the last dense layer, as the final output could be interpreted as the likelihood of being classified as each stage.

We also conducted traditional ROI-based machine learning methods in rs-fMRI analysis as the baseline. A standard pipeline is calculating the FC matrix, representing the correlation or covariance between each ROI pair (connectome), and then using the FC matrices as predictors in a traditional machine learning classifier (Dadi et al., 2019). Here, we tested random forest (RF), linear support vector machine classifier (SVC) (as in Abós et al., 2017; Zhang et al., 2019) and logistic regression (LR). The FC matrices were calculated using the Ledoit Wolf estimator (Ledoit and Wolf, 2004) for large covariance matrices, and only the upper triangle of the matrices were used as the inputs of the ML classifiers to prevent redundancy since the FC matrices are symmetric.

### 2.3. Model Training and Evaluation

To comprehensively evaluate the model performances, five repeats of 10-fold stratified cross validation was implemented. For each repeated run, 10% of subjects are selected as test set, 10% are selected as validation set, and the remaining 80% are the training set. Note that the random split was carried out on both stage 1 and stage 2 in order to stratify the imbalanced dataset and keep the ratio of the two stages roughly the same in all the splits. To handle the class imbalance, sample weights are assigned inversely proportional to the stage ratio in the dataset. For the LSTM model and all the ML models, the hyperparameter tuning was first performed by using the validation set to evaluate the performance of the models trained on the training set under the different hyperparameters. After the best hyperparameters were selected, the final model was trained on both the training and the validation set and then evaluated on the test set to best utilize the relatively smaller dataset.

For the LSTM model setup, the initial hidden and cell states of the LSTM were set as all zeros. The input length was set to 50 time points based on initial testing on a random cross-validation fold (details in Supplementary Material). The hidden size *M* of the LSTM layer was tuned from the list of [16, 32, 64, 128]. To prevent overfitting on the LSTM model, L2 regularization and the early-stopping mechanism were introduced to enhance the model's generalization ability. For both deep learning models, training was terminated when the validation loss had not reduced in 10 epochs or when the maximum epoch number 50 was reached. Both LSTM and CNN networks were trained using the cross entropy loss function, Adam optimizer with learning rate = 1e-4, batch size = 200, dropout rate fixed at 0.5, the λ of the L2 regularization at 0.01, and other default parameter settings. Both the LSTM and CNN models were implemented using PyTorch and trained on a NVIDIA GeForce GTX 1080 GPU at the Farnam Cluster of the Yale Center for Research Computing.

All the other ML models were implemented *via* Scikit-learn. The regularization parameter inversely proportional to the regularization strength of LR was searched in the range of [1e-9, 10] and the search range of SVC was [0.01, 10], both with step size as 1e-1 in log scale. For RF, the number of trees were tuned from the list of [10, 50, 100, 200, 500]. The maximum depth of the tree was searched from 2 to 6. The minimum number of samples to split an internal node was selected from the list of [10, 20, 50, 100, 200, 500, 1,000, 2,000, 3,000] and the searching list of the minimum number of samples in a leaf node was [10, 50, 100, 200, 300, 500]. All the other hyperparameters were set as default.

All the evaluation metrics were based on subject-level outputs, which are given by the majority vote of all the sequences from one subject. Evaluation at the subject level better matches the real clinical diagnosis of one label per subject, and the ensembling also has improved performance compared to the sample-wise results. The subject-wise accuracy, precision, recall (sensitivity), specificity, and F1 score were reported. The equations for accuracy, precision, recall, specificity, and F1 score calculation are as below,


(7) $$\begin{array}{l} \begin{array}{c} Accuracy=\frac{TP+TN}{TP+TN+FP+FN} \end{array} \end{array}$$

(8) $$\begin{array}{l} \begin{array}{c} Precision=\frac{TP}{TP+FP} \end{array} \end{array}$$

(9) $$\begin{array}{l} \begin{array}{c} Recall=\frac{TP}{TP+FN} \end{array} \end{array}$$

(10) $$\begin{array}{l} \begin{array}{c} Specificity=\frac{TN}{FP+TN} \end{array} \end{array}$$

(11) $$\begin{array}{l} \begin{array}{c} F1=\frac{2*Precision*Recall}{Precision+Recall} \end{array} \end{array}$$

where positive and negative refer to stage 2 and stage 1, respectively, *TP* is the true positive, *TN* is the true negative, *FP* is the false positive, and *FN* is the false negative.

The corrected repeated k-fold cross validation test (Bouckaert and Frank, 2004) was conducted as the significance test for model performance comparison. For a *r*-times *k*-fold cross-validation, the following statistic is calculated,


(12) $$\begin{array}{l} \begin{array}{c} t=\frac{\overset{k}{\underset{i=1}{\sum }}\overset{r}{\underset{j=1}{\sum }}x_{ij}}{\left(k*r\right)\sqrt{\left(\frac{1}{k*r}+\frac{n_{2}}{n_{1}}\right){\hat{\sigma }}^{2}}} \end{array} \end{array}$$

where *x*_*ij*_ is the difference in the statistic of interest between two models being compared from the *i*th fold of the *j*th cross-validation run, *n*_1_ is the number of subjects used for training, *n*_2_ is the number of subjects used for testing, and ${\hat{\sigma }}^{2}$ is the estimated variance of the differences *x*_*ij*_. This test corrects the estimate of the variance by taking the dependency between cross-validation samples into account. The significance level α was set at 0.05.

We also compared the model outputs to a separate measure of PD severity to further assess validity of model predictions for disease staging. The Movement Disorders Society-Unified Parkinson's Disease Rating Scale (MDS-UPDRS) is the standard assessment tool for disease severity and progression of PD (Goetz et al., 2008). The MDS-UPDRS part III motor exam score (range: 0–132) rates the severity of motor impairment. Higher scores indicate worse motor impairment. The MDS-UPDRS-III scores of stage 1 subjects are 14.00 ± 5.57, and that of stage 2 subjects are 23.69 ± 10.70. We computed the Pearson correlation between the model output scores and the MDS-UPDRS-III scores to evaluate whether the stage classification model output is associated with a more continuous measure of disease severity.

### 2.4. Model Interpretation and Connectivity Analysis

We interpreted the LSTM model by exploring the learnable input-hidden weights *W* of the LSTM cell (Dvornek et al., 2017). The ROIs associated with high magnitudes of the weights that are directly applied to the ROI time-series data play a significant role in giving the output, and thus will be given high importance in the model analysis. After z-score normalization, we highlighted the ROIs with the magnitudes of the associated weights above mean and one standard deviation of the weights. These ROIs were considered as the important ROIs for early stage PD classification.

Pairwise FC analysis was then conducted based on the selected top ROIs. The covariance matrices for all subjects were computed from the top ROIs. Then for each edge, the Welch's *t*-test (Ruxton, 2006) was used to compare the FC for the ROI pairs between the stage 1 and stage 2 subjects, taking into consideration the different number of subjects in each stage. The significance of the Welch's t-statistic was assessed using a permutation test with 10,000 random permutations of the subject stage labels to find whether there was a significant difference in the FC between the two stages. Correction for multiple comparisons was performed by controlling the false discovery rate (FDR; Benjamini and Hochberg, 1995).

Regression analysis was performed to analyze the association between the FC of top ROIs and the MDS-UPDRS-III scores. The elastic net model (Zou and Hastie, 2005) implemented using Scikit-learn was used to predict MDS-UPDRS-III scores from the pairwise FC between top ROIs. The elastic net regularization parameters were searched using the repeated cross-validation splitting strategy (3 runs, 10 folds), and the optimal hyperparameters were then applied to the model for the entire dataset. The searching range of both α and l1_ratio is [0, 1]. The selected regularization parameters for LSTM were α = 0.1936 and l1_ratio = 0.1000.

The permutation test with 10,000 runs was again used to assess significance of the regression coefficients, where the subject MDS-UPDRS-III scores were randomly shuffled and the *p*-values were calculated as the percentage of permutation results with a coefficient magnitude greater than the magnitude of the original observation. The top ROI analysis results were also compared with results of similar whole-brain FC analysis to see whether the top ROIs play a dominant part in disease stage progression. All the FC differences were visualized *via* BrainNet Viewer (Xia et al., 2013).

## 3. Results

### 3.1. Characterization Results

Table 3 summarizes the classification results of the LSTM model, the CNN model, and all the ML classifiers. All the models have been selected with the best hyperparameters. The proposed LSTM model yielded the highest values among all the quantitative metrics, outperforming the CNN model and all the other ML methods. Under the corrected repeated k-fold cross validation test (*k* = 10, *r* = 5, *n*_2_ = 8, *n*_1_ = 76, degree of freedom = 49, and *t*_*thre*_ = −2.010), the results of the LSTM model showed significant improvement in accuracy, F1, and recall compared to all other models. In precision and specificity, the LSTM model showed significant improvement compared to SVC.

**Table 3:** Early-stage PD classification results (mean ± standard deviation).

	**Accuracy**	**F1**	**Precision**	**Recall (Sensitivity)**	**Specificity**
LR	0.5664 ± 0.1897	0.6346 ± 0.2032	0.6993 ± 0.2089	0.6173 ± 0.2402	0.4666 ± 0.3605
RF	0.5862 ± 0.1510	0.6745 ± 0.1341	0.7199 ± 0.1548	0.6580 ± 0.1702	0.4433 ± 0.3155
SVC	0.5811 ± 0.1451	0.6508 ± 0.1721	0.7216 ± 0.1930	0.6373 ± 0.2253	0.4766 ± 0.3496
CNN	0.6007 ± 0.1399	0.6939 ± 0.1549	0.6952 ± 0.1435	0.7293 ± 0.2240	0.3266 ± 0.2807
LSTM	**0.7163** **±0.1318**	**0.7912** **±0.1050**	**0.7794** **±0.1047**	**0.8226** **±0.1579**	**0.5833** **±0.2948**

*The best results were marked in bold*.

Table 4 presents the correlation between the model output scores and the MDS-UPDRS-III scores. The SVC model, which resulted in the second highest precision and specificity for stage classification (Table 3), did not produce significant correlation of model output and MDS-UPDRS-III scores (*r* = 0.1748, *p* = 0.12). The LSTM model showed the highest correlation between the stage prediction score with MDS-UPDRS-III scores (*r* = 0.4270, *p* = 0.00003).

**Table 4:** Correlation between the model output scores and the MDS-UPDRS-III scores (mean ± standard deviation of 5 runs).

	**Pearson correlation**
LR	0.3212 ± 0.0550
RF	0.3911 ± 0.0231
SVC	0.1748 ± 0.0455
CNN	0.2686 ± 0.0230
LSTM	**0.4270** **±0.0595**

*The best result was highlighted in bold*.

### 3.2. Brain Abnormality Detection

We detected the top ROIs related to the brain abnormality in disease development by investigating the learnable weights in the LSTM model. Table 5 displays the top ROIs with the greatest absolute weights for the overall LSTM model in descending order. The level is the computed z-score of the absolute value of the extracted weights, showing how much the region magnitude is above the mean weight magnitude of all the ROIs.

**Table 5:** Top ROIs and the associated brain functions with the greatest weight magnitudes in the LSTM model.

**Region**	**Level**	**Function**
Vermis_10	2.4741	Motor functions
Inferior frontal gyrus, orbital part, right	2.2505	Higher cognitive functions
Calcarine sulcus, right	1.9351	Visual functions
Middle frontal gyrus, orbital part, left	1.8824	Higher cognitive functions
Insula, right	1.8568	Emotional functions
Calcarine sulcus, left	1.8407	Visual functions
Middle frontal gyrus, orbital part, right	1.8824	Higher cognitive functions
Caudate nucleus, right	1.7248	Motor functions
Superior occipital gyrus, right	1.5491	Visual functions
Superior frontal gyrus, medial, left	1.5404	Higher cognitive functions
Amygdala, left	1.5041	Emotional functions
Postcentral gyrus, right	1.4804	Somatosensory functions
Cerebellum_3, left	1.4733	Motor functions
Posterior cingulate gyrus, right	1.4275	Default mode functions
Supplementary motor area, left	1.3535	Motor functions
Cerebellum_7b, right	1.4733	Motor functions
Lenticular nucleus (putamen and globus pallidus), right	1.1469	Motor functions
Cerebellum_6, right	1.4733	Motor functions
Superior occipital gyrus, left	1.0606	Visual functions

### 3.3. Brain Connectivity Analysis

**Figure 3:**
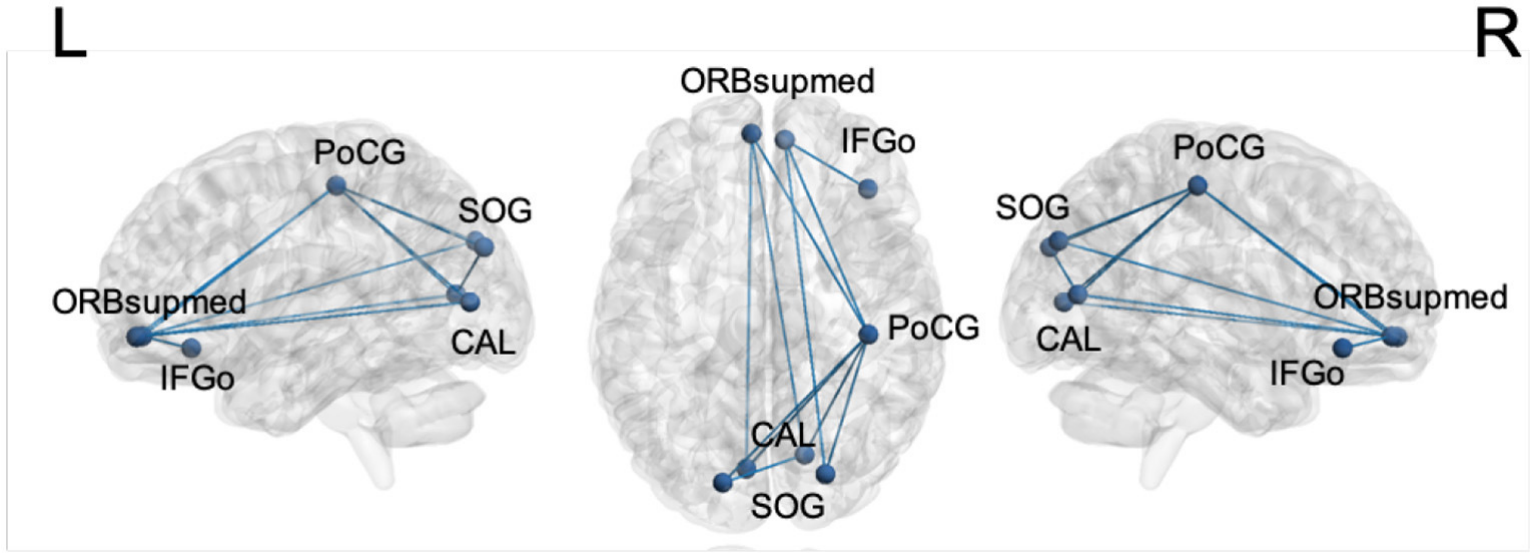
Significantly different edges in stage 1 and 2 by permutation Welch's *t*-test for the LSTM model. All edges showed decreased connectivity in stage 2. PoCG, postcentral gyrus; SOG, superior occipital gyrus; CAL, calcarine sulcus; ORBsupmed, orbitofrontal cortex, superior medial part; IFGo, inferior frontal gyrus, orbital part.

The brain connectivity analysis was carried out first by assessing FC differences between stages 1 and 2 for all the pairwise connections between the top ROIs by using the permutation Welch's *t*-test. The edges that showed significant differences between the two stages are listed in Table 6, and the nodes and edges are displayed in Figure 3. All listed edges were weaker in stage 2 than in stage 1, indicating that a more advanced disease stage in PD is associated with decreased functional connectivity.

**Table 6:** Significantly different edges in stage 1 and 2 by permutation Welch's *t*-test for the LSTM model.

**Edges connecting ROIs**	***P*-value**
Postcentral gyrus, right-Superior occipital gyrus, left	0.0022
Calcarine sulcus, right-Superior occipital gyrus, left	0.0077
Inferior frontal gyrus, orbital part, right- Middle frontal gyrus, orbital part, right	0.0093
Superior occipital gyrus, right-Postcentral gyrus, right	0.0096
Middle frontal gyrus, orbital part, right-Postcentral gyrus, right	0.0159
Calcarine sulcus, right-Postcentral gyrus, right	0.0201
Middle frontal gyrus, orbital part, left-Postcentral gyrus, right	0.0275
Calcarine sulcus, right-Middle frontal gyrus, orbital part, left	0.0287
Calcarine sulcus, left-Postcentral gyrus, right	0.0310
Middle frontal gyrus, orbital part, left-Calcarine sulcus, left	0.0327
Middle frontal gyrus, orbital part, right-Superior occipital gyrus, right	0.0329

**Figure 4:**
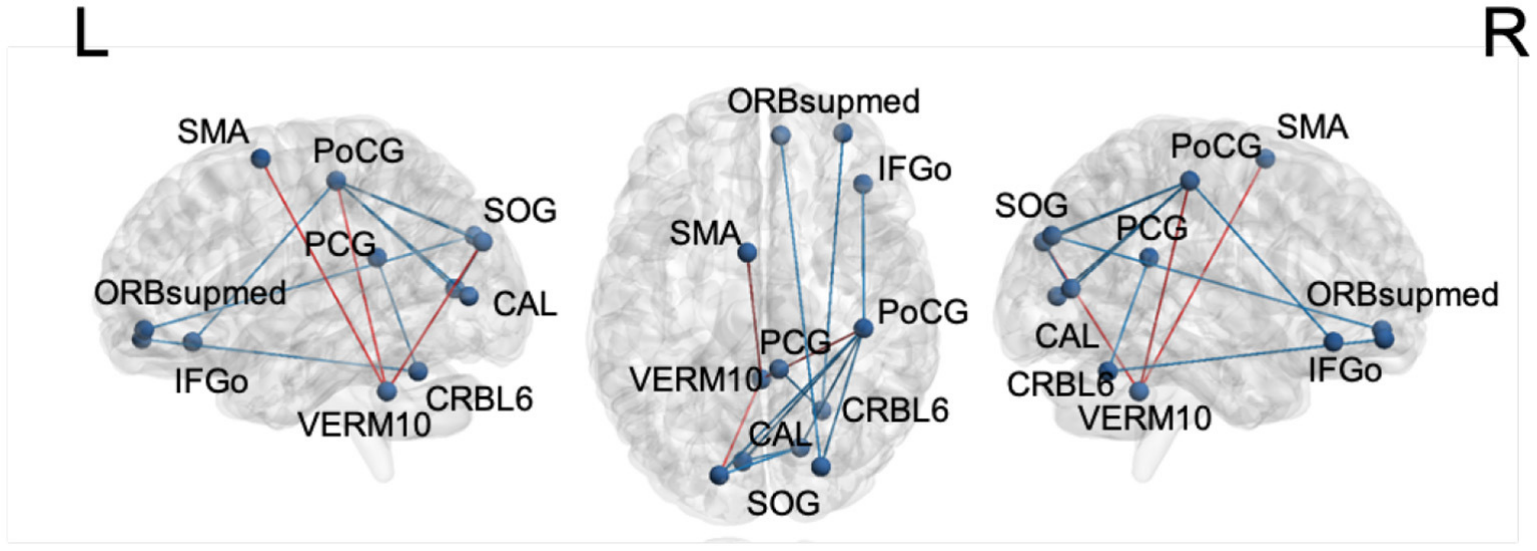
The edges with significant weights by permutation *t*-test of elastic net regression of MDS-UPDRS-III score for the LSTM model. Red: edges with positive coefficients. Blue: edges with negative coefficients. SMA, supplementary motor area; PoCG, postcentral gyrus; SOG, superior occipital gyrus; PCG, posterior cingulate gyrus; CAL, calcarine sulcus; ORBsupmed, orbitofrontal cortex, superior medial part; IFGo, inferior frontal gyrus, orbital part; VERM10, vermis_10; CRBL6, cerebellum_6.

For motor impairment-related connectivity analysis, the elastic net regression was used to regress the MDS-UPDRS-III score on the FC edges and permutation testing was conducted to assess the significance of regression coefficients. Table 7 summarizes the significant edges of the regression results and the related direction of association with motor score. The nodes and edges are displayed in Figure 4. After applying the FDR correction with a false discovery rate of 0.2, the top three edges remained significant.

**Table 7:** Edges with significant weights by permutation test of the elastic net regression of MDS-UPDRS-III scores for the LSTM model.

**Edges connecting ROIs**	***P*-value**	**Direction**
Calcarine sulcus, left-Postcentral gyrus, right	0.0004	Decreased
Calcarine sulcus, right-Postcentral gyrus, right	0.0005	Decreased
Superior occipital gyrus, right-Postcentral gyrus, right	0.0019	Decreased
Superior occipital gyrus, left-Postcentral gyrus, right	0.0089	Decreased
Calcarine sulcus, left- Calcarine sulcus, right	0.0106	Decreased
Vermis_10-Superior occipital gyrus, left	0.0217	Increased
Middle frontal gyrus, orbital part, right-Superior occipital gyrus, right	0.0233	Decreased
Posterior cingulate gyrus, right-Cerebellum_6, right	0.0234	Decreased
Vermis_10-Postcentral gyrus, right	0.0238	Increased
Inferior frontal gyrus, orbital part, right-Postcentral gyrus, right	0.0243	Decreased
Middle frontal gyrus, orbital part, right-Cerebellum_6, right	0.0258	Decreased
Vermis_10-Supplementary motor area, left	0.0325	Increased
Calcarine sulcus, right-Superior occipital gyrus, left	0.0434	Decreased

A conventional whole-brain FC permutation Welch's *t*-test and MDS-UPDRS-III score regression analysis were conducted as a comparison with the sub-ROI group analysis (details in Supplementary Figures 1, 2, respectively). Note that none of the edges detected by whole-brain analysis survived the FDR correction, potentially indicating overfitting in the traditional whole-brain analysis results.

## 4. Discussion

In this work, we investigated an LSTM model for early-stage PD characterization using rs-fMRI data. Under a 5-run, 10-fold repeated stratified cross-validation, the proposed LSTM model performed significantly better than the CNN model and the other traditional ML methods for the classification of stage 1 and stage 2 PD subjects. The model output scores were also better correlated with the motor severity scale. The learnable weights in the well-trained LSTM model produced meaningful interpretations. The *post-hoc* FC analysis revealed edges that differed significantly between stages 1 and 2 in both classification and regression analyses. The findings of potentially influential top brain regions and abnormal FC among them could provide a deeper understanding of the neuroanatomical substrates of early disease stages in PD.

The classification results suggest the LSTM model has the potential to better extract and utilize temporal information from the rs-fMRI data. The conventional ML analysis methods are FC-based, i.e., the FC matrices are the input of the ML methods instead of the original rs-fMRI series. This requires an additional step of data pre-processing, which needs additional time and computation, while it may also cause some loss in the functional information. Our proposed method directly uses the rs-fMRI series as the input, which successfully preserved brain functional information while reducing noise and redundancy. While both the LSTM and CNN models used the rs-fMRI series as input, the LSTM model performed better than the CNN model. The input gate, forget gate, and output gate in the LSTM cell are designed to handle temporal dependencies in relatively long sequences, which could successfully extract temporal information along the time dimension in a time- and computation-efficient way.

Furthermore, the LSTM model outputs produced the highest correlation with the MDS-UPDRS-III scores. A high correlation is desired, as the degree of motor impairment plays an important part in early stage distinction. Thus, the proposed LSTM model not only resulted in the best stage classification performance, but also the confidence of the LSTM model's classification produced the highest correlation with a closely related continuous rating of disease severity.

In brain abnormality detection, a diverse set of ROIs were important for predicting disease severity. Most notably, the basal ganglia structures including the lenticular nucleus and caudate, and the supplementary motor area together with the postcentral gyrus are implicated in sensorimotor impairment in PD (Ji et al., 2018). The cerebellum is involved in tremor generation (Ma et al., 2015; Zhang et al., 2016). The amygdala and insula are limbic structures involved in emotional processing and play a role in anxiety and depression in PD (Tinaz et al., 2021). The calcarine sulcus and superior occipital gyrus are visual processing areas implicated in visual symptoms of PD such as hallucinations (Bejr-kasem et al., 2019). The posterior cingulate is the major hub in the default mode network that shows abnormal FC in PD and other neurodegenerative disorders (Tinaz, 2021). Finally, the frontal regions mediate higher cognitive and executive functions and are implicated in cognitive dysfunction in PD even in the early stages and in the absence of dementia (Tinaz, 2021). Thus, the top influential ROIs for the LSTM model that play an important role in distinguishing early-stage PD are also relevant brain regions linked to motor and non-motor functions that are affected in PD.

In brain connectivity analysis, interestingly, the influential edges are mostly between the nodes related to non-motor brain functions. This finding has important clinical implications suggesting that progression even in the early stages of the disease involves FC changes in non-motor networks, underlining the importance of evaluating the severity of not only motor but also non-motor impairment in clinical progression studies and prediction models (Tinaz et al., 2021). Current clinical works investigating non-motor neuropsychiatric symptoms in PD (Alzahrani et al., 2015) and the related FC analyses (Baggio et al., 2015) supports this finding as well.

In motor impairment-related connectivity analysis, higher MDS-UPDRS-III scores indicate worse motor impairment. Therefore, the “increased” direction in Table 7 denotes a positive relationship between edge strength and motor impairment, whereas “decreased” shows the opposite relationship. The edge strength between the cerebellar vermis and sensorimotor (supplementary motor area and postcentral gyrus) and visual areas (superior occipital gyrus) is associated with worse motor impairment suggesting an reorganization of brain circuits. Similar shifts from the defective basal ganglia circuits to the cerebellar circuits have been reported in PD (Tinaz, 2021).

The brain connectivity analysis of the LSTM model was based on the top ROIs with high magnitudes of the learned weights, which showed its superiority compared with the conventional whole-brain FC analysis. While the results of the LSTM ROI analysis showed similar trends as the traditional whole-brain FC analysis in terms of highlighting similar regions with increased or decreased connectivity, the very large number of connections in the whole-brain FC analysis not only hinders interpretation but also did not survive correction, thus, were not informative. Furthermore, given the relatively smaller number of subjects, the whole-brain FC analysis could result in overfitting. Thus, these analyses may not be robust in revealing FC changes that generalize to the greater PD population. The *post hoc* regression results of the LSTM model using the FC between the top ROIs also highlighted the connections that may be implicated in disease severity, whereas the traditional FC analysis did not show a significant relationship between disease severity and whole-brain FC across the entire group. In conclusion, the LSTM model was able to accurately classify the two early disease stages by identifying the specific brain regions and edges that contribute strongly to disease stage and motor impairment.

There are some limitations of this work that require further investigation. First, we applied a single pipeline for the pre-processing of the rs-fMRI data without global signal regression (GSR). Other pre-processing strategies including GSR could reveal different FC results (Murphy et al., 2017). As there is not a single right way of pre-processing rs-fMRI data (Murphy et al., 2017), a future direction of this study would be investigating the effect of other pre-processing strategies such as GSR on the FC results in PD early stage characterization. Also, the current work focuses on the usage of only rs-fMRI data in early stage PD classification. Future work should introduce multimodal data into the characterization, such as task-based fMRI data, cognitive and behavioral assessments, and other biological markers. This will fully utilize each patient's clinical profile and more comprehensively assess factors that may predict disease stage, potentially improving stage classification and thus producing more fruitful features for characterizing differences between the two early stages. Another promising future direction is investigating the usage of other advanced temporal deep learning models such as gated recurrent unit (Cho et al., 2014) and transformer (Vaswani et al., 2017) networks. By implementing various network backbone structures, the classification performance is expected to further improve with insightful biomarker findings.

## 5. Conclusion

We proposed the usage of an LSTM model for early stage PD characterization using rs-fMRI data from the majority of the PPMI dataset. Under the repeated stratified cross-validation, the LSTM model significantly outperformed the CNN model and other ML methods in accuracy, F1 score, and recall, with the highest correlation between model output scores and the MDS-UPDRS-III motor scores. The LSTM model interpretation results suggested the highly influential ROIs in early stage PD progression, and the brain connectivity analysis results identified prominent edges in brain FC changes and the disease severity. The propounded regions and edges are related to the symptoms of PD, which supports the validity of our proposed LSTM model for early stage characterization. Identification of brain regions and functional connections affected by early PD progression could potentially help unravel the mechanisms of PD and facilitate the development of new therapeutic targets.

## Data Availability Statement

The data analyzed in this study was obtained from Parkinson's Progression Markers Initiative (PPMI), the following licenses/restrictions apply: Investigators seeking access to PPMI data must sign the Data Use Agreement, submit an Online Application and comply with the study Publications Policy. Requests to access these datasets should be directed to PPMI, https://www.ppmi-info.org/access-data-specimens/download-data.

## Ethics Statement

The original PPMI study was conducted following the Declaration of Helsinki and the Good Clinical Practice (GCP) guidelines approved by the Local Ethics Committees of the 24 participating sites in the US (18), Europe (5), and Australia (1) with informed consent obtained from all the enrolled subjects. The patients/participants provided their written informed consent to participate in this study.

## Author Contributions

XG: conceptualization, methodology, software, validation, formal analysis, visualization, and writing—original draft, review, and editing. ST: conceptualization, data curation, and writing—review and editing. ND: conceptualization, methodology, writing—review and editing, and supervision. All authors contributed to manuscript revision, read, and approved the submitted version.

## Funding

XG was supported by the Biomedical Engineering Ph.D. fellowship at Yale University. ST was supported by the National Institute of Neurological Disorders and Stroke (Grant Number K23NS099478).

## Conflict of Interest

The authors declare that the research was conducted in the absence of any commercial or financial relationships that could be construed as a potential conflict of interest.

## Publisher's Note

All claims expressed in this article are solely those of the authors and do not necessarily represent those of their affiliated organizations, or those of the publisher, the editors and the reviewers. Any product that may be evaluated in this article, or claim that may be made by its manufacturer, is not guaranteed or endorsed by the publisher.
