# Supplementary material for: Characterization of Early Stage Parkinson's Disease From Resting-State fMRI Data Using a Long Short-Term Memory Network
Source: Front Neuroimaging. 2022 Jul 13;1:952084. doi: 10.3389/fnimg.2022.952084 (PMC10406199; doi:10.3389/fnimg.2022.952084)
Supplement: Supplementary file 1 [file Data_Sheet_1.pdf]

Supplementary Material

1 INPUT SEQUENCE LENGTH TESTING

The rs-fMRI sequence length selection was based on an initial test on a random fold of the stratified cross-validation. The length was tuned in the range of [10, 50, 100], representing 24 seconds, 2 minutes, and 4 minutes of scanning, respectively. As in Supplementary Table S1, the setting of sequence length = 50 achieved the highest sample accuracy, F1 score, and sensitivity, while length = 10 achieved the highest specificity. Based on the overall evaluation, it is potentially suggested that if the sequence length is too short, the lacking of temporal information could lead to weakness in distinguishing the two stages; however, if the sequence length is too long, the total number of samples after augmentation would be smaller, and the provided temporal information would also potentially be too redundant since rs-fMRI series can be relatively noisy.

2 WHOLE-BRAIN FUNCTIONAL CONNECTIVITY ANALYSIS

A conventional whole-brain analysis was performed to determine FC differences between stage 1 and stage 2 on the 6,670 edges in total. Similarly to the LSTM ROI analysis, significance of the FC differences were assessed using the permutation test of the Welch's t-test, conducted on all the edges of the whole brain ROIs with 10,000 random permutations. For each edge, the significance of the Welch's t-statistic was assessed to compare whether there was a significant difference in the FC for the ROI pair between the stage 1 and stage 2 subjects. FDR correction was also applied for the large number of multiple comparisons. The significantly different edges of the whole-brain analysis (uncorrected) are visualized in Figure S1, yet none of the detected edges survived the FDR correction with the same false discovery rate of 0.2 as in the top ROI analysis. This potentially indicates that the whole-brain analysis results in overfitting and might not be reliable in revealing FC changes related to the disease stage.

An elastic net regression model was also estimated to regress the MDS-UPDRS-III motor scores onto the set of whole-brain connections with the regularization parameters set to α = 0.1744 and l1_ratio = 1.0000. The searching range of both α and l1_ratio is [0, 1], the same as the regression analysis for LSTM model interpretation. The optimal parameters were selected under the same repeated cross-validation splitting strategy (3 runs, 10 folds) as the top FC analysis. Similar to the LSTM ROI analysis, significance of the regression coefficients was assessed using the permutation test. Permutation testing with 10,000 runs was conducted and the p-values were calculated as the percentage of permutation results with a coefficient magnitude greater than the magnitude of the original observation. In Figure S2, the edges with significant weights for the whole-brain regression are visualized. Note that none of the detected edges were significant after applying the same 0.2 threshold for FDR correction.

Table S1: The effect of sequence length.

Sequence length	Sample accuracy	Sample F1	Sample sensitivity	Sample specificity
10	0.702	0.719	0.766	0.639
50	0.728	0.758	0.839	0.615
100	0.571	0.510	0.566	0.538

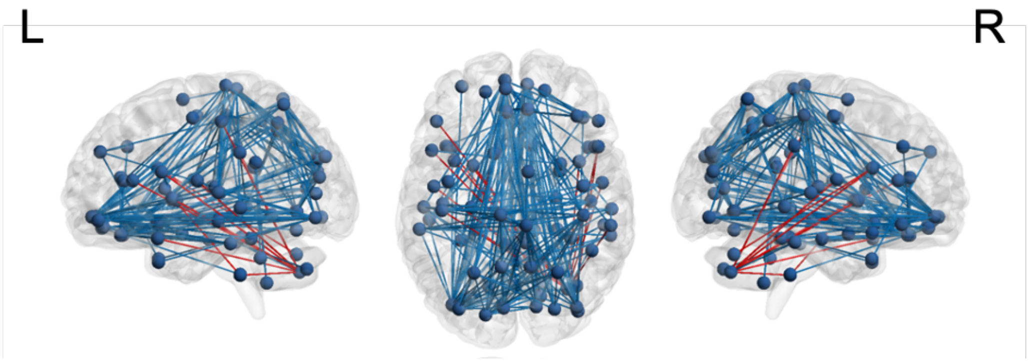

Figure S1: The significantly different edges in stage 1 and 2 by permutation Welch's t-test for the whole brain analysis. Red: increased edges. Blue: decreased edges.

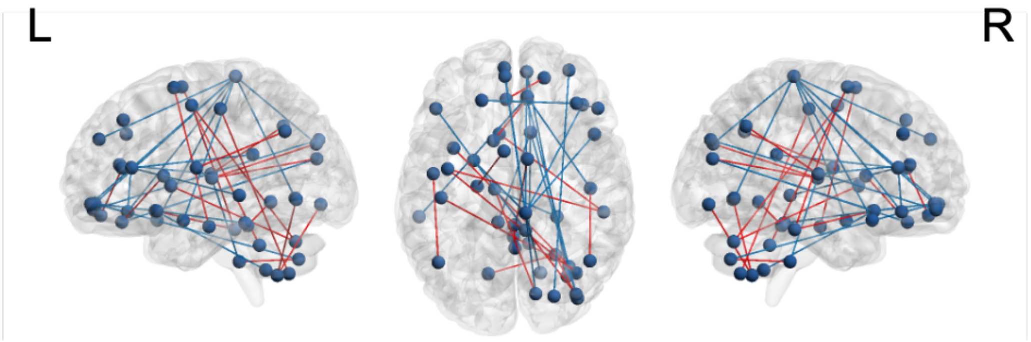

Figure S2: The edges with significant weights by permutation t-test of elastic net regression of MDS-UPDRS-III score for the whole brain analysis. Red: increased edges. Blue: decreased edges.
